# University of London

**Published:** 1864-04

**Authors:** 


UNIVERSITY OF LONDON.—Her Majesty has nominated the Right Hon. Edward Cardwell, M.P., and Dr. William Sharpey, secretary to the Royal Society, to be members of the Senate of the University of London.

